# Supplementary material for: Chromothripsis during telomere crisis is independent of NHEJ, and consistent with a replicative origin
Source: Genome Res. 2019 May;29(5):737–49. doi: 10.1101/gr.240705.118 (PMC6499312; doi:10.1101/gr.240705.118)
Supplement: Supplemental Material [file supp_gr.240705.118_Supplemental_file_1.zip › contigs/annotated_contigs/DB76/contig.2.DB76_length_379_mean_cov_20.018469657.docx]

**DB76_length_379_mean_cov_20.018469657**

```
CTACTAAGCCAAAATACAAAAAATTAGCCAGGCGTGGTGGCGGGCGCCTGTAGTCCCAGCTACTCAGGAGGTTGAGGCAGGAGAATGTT
 >chr15:30373566-30373793 + E=5e-112
GTGAACCCGGGAGGCGGAGCTTGCAGTGAGCCAAGATCGCGCCACTGCACTCCAGCGTGGGTGACAGAGCGAGACTCCATCTCAAACAA

AAAAAAAAAAATGAACATGTCATCCATACTTCTAAGGTGTT|TTG|AAGGATCAATGCAACAAGAAGAGCTTACTATCCTAAATATATG
                                             >chr15:30313327-30313484 + E=5e-83
TGTGCTCAATATAGGAGCACCCAGATTCATAAAACAAGTTCCTAAAGACCTATAAAGAGACTTAGACCCCCAAATAGTAATAGTGGGAG

ACTTTAACACCCCACTGTCAATATT
```
